# Why service users do not complain or have ‘voice’: a mixed-methods study from Nepal’s rural primary health care system

**DOI:** 10.1186/s12913-017-2034-5

**Published:** 2017-01-25

**Authors:** Gagan Gurung, Sarah Derrett, Robin Gauld, Philip C. Hill

**Affiliations:** 1 Department of Preventive and Social Medicine, Dunedin School of Medicine, PO Box 56, Ground Floor, Adams Building, 18 Frederick Street, Dunedin, 9016 New Zealand; 2 Injury Prevention Unit, Department of Preventive and Social Medicine, Dunedin School of Medicine, Dunedin, New Zealand; 3 Otago Business School, University of Otago, Dunedin, New Zealand; 4 Centre for International Health, Department of Preventive and Social Medicine, Dunedin School of Medicine, Dunedin, New Zealand

**Keywords:** Service users, Patients, Complaints, Voice, Primary health care, Nepal

## Abstract

**Background:**

Despite abundant literature on the different aspects of health care complaint management systems in high-income countries, little is known about this area in less developed health care systems and most research to date has been conducted in hospital settings. This article seeks to address this gap by reporting on research into complaint systems in primary health care (PHC) settings in Nepal.

**Methods:**

Using a mixed-methods design, qualitative interviews were conducted with key informants (*n* = 39) and six community focus groups (*n* = 56), in the Dang District of Nepal. In addition, interviewer-administered structured questionnaire interviews were held with 400 service users, health facility operation and management committee (HFMC) members and service providers from 22 of the 39 public health facilities. Qualitative data were transcribed, organized and then analyzed using the framework method in QSR NVivo 10, while quantitative data were analyzed using IBM SPSS 22.

**Results:**

Despite service users having grievances with the health system, they did not complain frequently: 9% (*n* = 20) reported ever making complaints about the PHC services. Complaints made were about medicines, health facility opening hours, health facility physical environment, and service providers, and were categorized into environment/equipment, accessibility/availability, level of empathy in the care process and care/safety. Generally, complaints were made verbally to health providers or to HFMC members or female community health volunteers. Use of formal channels such as suggestion boxes or written complaints was almost non-existent. Reasons reported for not complaining included: a lack of complaint channels; lack of knowledge of service entitlements; power asymmetry between service providers and service users; lack of opportunity to choose alternative providers, lack of an established culture of complaining, and a perceived lack of responsiveness to complaints.

**Conclusion:**

Very few service users made complaints to PHC services in Nepal. Several contextual factors related to the community and the health system were identified as the reasons for not complaining. We recommend continuing efforts to establish proper complaints mechanisms with an increased emphasis on the existing community health system networks. Furthermore, awareness among service users about service entitlements and complaint mechanisms should be increased.

## Background

It is increasingly recognized that patients as consumers of health care services, can provide critical feedback and valuable information to health care system by voicing their complaints and suggestions [1, 2]. Many countries such as the United Kingdom, have introduced health service reforms with introduction of commercial elements and market influences in the health sector in the 1980’s which gave rise to patients’ complaints as a management tool to improve health service accountability and responsiveness [2–4]. Indeed, the ideology guiding the patients’ complaints mechanisms can be linked to the increasing trend of consumerism in health care [4].

Service users’ complaints (sometimes referred to as suggestions, feedback, or grievances) can reflect health service failures and deficiencies and can provide valuable insights into services by identifying problem areas which management may not be aware of [5, 6].

For effective utilization of complaints data, there needs to be a good complaints system which facilitates systematic ways to collect, analyze and respond to complaints [7]. There are different rationales for setting up a complaints system: to provide supplementary information to monitor the quality of care; to improve patient safety by identifying medical errors; and to improve and regulate the practice of health professionals [6, 8–10]. Another reason for complaints systems is to address corruption in the health sector [7]. Corruption in the form of informal payments has been found to be high in many low-and-middle income countries (LMICs), including Nepal [11, 12] and a sound patient complaints system potential to reduce this practice [7].

With increasing awareness of the value of patients’ complaints in understanding and improving health services, there is a growing body of research examining healthcare complaints [6, 13, 14]. To date, many studies have been conducted in high-income countries such as the United Kingdom, Australia, United States, Taiwan, New Zealand, France and Finland [2, 6] and, more recently, in some middle-income countries such as China and Vietnam [7, 15]. These studies have tended to focus on the different aspects and features of complaints: the complaint numbers/frequency; complainant and respondent profiles, nature, types, outcomes and reasons for complaints; barriers to complaining; and the impact of complaint management programs [2, 9, 14, 16–21]. For example, studies have found that the number of patient complaints is rising internationally [9, 22]. Prior studies have also reported on the nature and types of complaints [2, 20] highlighting the difficulties associated with developing a systematic classification of complaints [6, 21]. Recent studies have attempted to provide a taxonomy of patients’ complaints to help analyze complaints data [2, 6, 13]. For example, a study conducted in Taiwan in a hospital setting categorized complaints into care/treatment, communication, humaneness (respect, dignity, and staff attitudes), access/availability, environment/equipment, business practice and billing and payment [2].

Studies in high-income countries, have found complaint management systems are usually in place; however, countries tend to have different types of complaints systems [2, 10, 15, 20, 23] and different regulations [7]. Despite these differences, the complaints channels generally include local and national authorities within the health system and outside the system including ombudsmen and the courts [7, 10]. Furthermore, different countries have tested different tools for complaining, both formal and informal, verbal and written [15]. These include suggestion boxes, websites, notice boards, letters or emails, face-to-face meetings, a telephone hotline, and the patient council meetings [2, 7, 15, 24].

As mentioned, much research to date has focused on complain systems in high-income countries, and research also tends to have focused on complaints in hospital settings. Research has focused on patient satisfaction in LMICs, a traditional measure of quality of care, but patient satisfaction as a measure of quality has been criticized for not accurately and fully capturing the patients’ voice [1, 2]. Little is currently known about complaint management systems in LMICs [15]. Most importantly, it is important to explore the relevance of the concept of patients’ complaints as a management tool, which has origins in high-income countries, in LMIC settings, with their different historical and cultural health systems contexts.

This article reports on research into complaint systems conducted in primary health care (PHC) settings in Nepal – a LMIC.

## Nepal’s PHC system context

Primary care, the main component of Nepal’s health care system, provides essential services to most of the population [25], and includes a network of nearly 4000 peripheral health facilities (sub-health posts, health posts, and PHC centers) [26]. These provider facilities are managed and supported by district (public) health offices [27]. Community-based services are provided by female community health volunteers (FCHVs), immunization clinics, and outreach clinics [26]. FCHVs are local women, selected by mothers’ groups in villages; they conduct monthly meetings with their groups and provide community health services [27, 28]. All PHC facilities have a health facility operation and management committee (HFMC) to manage funds, human resources, and health programs locally [29, 30]. Each HFMC consists of 9 to 13 representatives from the villages, and the membership includes the manager of the health facility, the village development committee chairperson and elected members including school teachers, FCHVs, Dalit (a marginalized caste), Janajati (an ethnic group) and female members [29, 30].

Despite the many challenges (natural, political and socioeconomic), Nepal has achieved impressive health gains in recent decades [27] but quality is a serious challenge facing Nepal’s primary health care system [31]. Perceived quality has been identified as a factor influencing utilization or bypassing of health services [32, 33]. While there have been recent increases in the utilization of services, attendance at primary care clinics by the population remains relatively low in Nepal [34]. Hence, there is potential for effective complaints management processes to improve health care quality and to make health systems more responsive and accountable to the community.

Recognizing the importance of service users’ feedback for service improvement, in 2008 Nepal’s Government introduced legislation requiring all public service delivery institutions to establish appropriate complaints structures and provision of a Citizen’s Charter [35, 36]. In the public health sector, the government also required mechanisms such as the HFMCs, suggestion boxes, and the Citizen’s Charter to be located in all provider facilities. In the absence of information about complaint systems in PHC contexts of Nepal, this study aimed to: 1) describe whether or not, and ‘how’, Nepali patients and public complained; 2) identify the main types of complaints/concerns raised; and 3) identify barriers to raising concerns about health services in Nepal with a view to informing improved complaint processes and systems.

## Methods

### Study design

This article presents findings from a mixed-methods study in the Dang District of Nepal conducted in 2014/2015. The district is located 280 km west of Kathmandu, Nepal’s capital [37]. Ecologically, the district has a diverse topography (hill and plain) and ethnic composition. It has an estimated population of 552 583, of which nearly 80% live rurally; two thirds are dependent on agriculture [37]. The district health system contains networks of 39 PHC health facilities, which include 21 sub-health posts, 15 health posts, and three PHC centres, managed by the Dang District Public Health Office [38].

### Data collection

The study included both qualitative and quantitative components. A quantitative structured face-to-face interview questionnaire was used to support the main qualitative component of the study. Following pre-testing, questionnaires were administered by interviewers to 400 participants (220 service users, 100 HFMC members and 80 service providers) from 22 of 39 public health facilities in the Dang District. Three separate, but similar, interviewer-administered structured questionnaires were used among three groups of participants: service users, HFMC members, and health facility service providers. Stratified random sampling was used to select public health facilities of the Dang district. The health facilities were stratified by PHC center, health posts, and sub-health posts. Then, 22 health facilities (two PHC centers, 11 health posts, and nine sub-health posts) were selected from each strata using simple random sampling. The selection of 22 out of 39 health facilities was necessary due to resource and time constraints. All of the service providers and HFMC members (service providers: *n* = 114; committee members: *n* = 219) of the 22 health facilities were requested to participate. In the case of service users, service user flow at each sampled health facility was calculated, and the number of the service users to be interviewed per health facility was determined by applying probability proportional to size. Then, the required sample per health facility was selected using systematic sampling. The questionnaire focused on the incidence, types, and methods of complaining.

The qualitative component explored different aspects of service users’ complaints to the PHC system and factors affecting complaining. Thirty-nine qualitative interviews were conducted using open-ended interviewing techniques. Interviews were undertaken with HFMC members, service providers, district level health managers and non-government organization (NGO) members; six focus groups were also held with 56 general community residents. A qualitative interview guide included a list of topics and questions to be covered. However, the interview process was flexible giving interviewees freedom to discuss topics of greatest importance and relevance to them. All 39 interviews and six focus groups were conducted by the lead author. A research assistant helped by recording detailed field notes for 30 interviews and all focus groups.

### Data analysis

All qualitative interviews and focus groups were audio taped, transcribed, and analyzed using NVivo 10 [39]. The framework method of analysis was used [40, 41], involving: transcription, familiarization with the data, organizing data within NVivo, coding, identification of an analytical framework, indexing and sorting, summarizing and displaying data and, finally, interpreting the findings. Pre-coded quantitative data from the structured questionnaires were entered, checked for data quality, and analyzed using IBM SPSS Statistics 22 [42] for descriptive analyses.

## Results

### Characteristics of participants from the questionnaire surveys

Overall, 220 service users, 100 HFMC members and 80 service providers participated (*N* = 400). More than two-thirds (68%) of service users were attending for curative services. A majority were female (66%) with a mean age of 33.7 years, ranging from 19 to 81 years. The main caste/ethnicity of the service users was Janajati (an ethnic group) (42%), followed by upper caste (36%) which is very close to the caste/ethnicity distribution in the district. More than 60 percent of the HFMC members were male (62%), and upper caste (63%) with the mean duration of service being 4.3 years. In the case of service providers, a majority of the respondents were permanent workers (64%), female (55%) and upper caste (69%); the mean duration of service for service providers was 11.5 years.

### Characteristics of participants from the qualitative interviews and focus groups

Of the 39 individual qualitative interview participants, 34 were male and 5 were female. Of the total respondents, the majority were Brahman/Chhetri (upper caste) followed by Dalit (a marginalized caste) and Janajati (an ethnic group). There was participation from community, health facility and district levels. Most of the participants were HFMC members (15), followed by service providers/managers (14), and NGO staff/members (10). Out of the six focus groups conducted, five were with community members (one male group, 4 female groups), and one with FCHVs. A total of 56 participants made up the 6 groups (8 male, 48 female), with each group consisting of participants ranging from eight to 13 in number. Among the female groups, most were affiliated with community mothers’ groups and users of health services provided by the public health facilities; two groups were from nearby health facilities and two were from relatively remote areas. The male group members were ordinary citizens affiliated with different professions and also users of health services from the nearest public health facilities.

### Did service users complain?

It was found that service users had grievances with, and/or suggestions for, the PHC system, but they did not complain frequently. The questionnaire survey found only 9% (*n* = 20) of service users reported ever making complaints to authorities. Likewise, 82% of HFMC members and service providers (*n* = 82 and 66 respectively) reported that service users made complaints only very occasionally.

Qualitative interviews identified that many service users grumbled with fellow community members rather than complaining to health providers. Further, they made complaints only with respect to a problem perceived as severe. Generally, those who were educated and financially secure complained, but the general public, especially the poor and Dalit (a marginalized caste) did not tend to do so:

‘There are grievances with people about the health post services, but they do not complain generally. They do so only very occasionally when it is very severe, and the complaints are mostly from those who are a little educated and empowered. Ordinary people, poor and Dalit cannot make complaints’ (Qualitative interview, HFMC member1, health post3).

‘Many people do have complaints but do not express these to the service providers. But they share their dissatisfaction with others in the community. I call it hidden complaints. You know, the bad things spread quickly which ultimately affects the reputation of the health post. And it increases the mistrust between service providers and service users’ (Qualitative interview, NGO member, health post1).

### Types of complaints

When service users did make complaints, these were for a range of reasons. From analysis of the open ended responses to the questionnaire (among those service users who ever made complaints, *n* = 20) and also from the qualitative interviews, the most common complaints made by service users were related to medicines, service providers, and health facility opening hours. Types of complaints fell into four categories: environment/equipment, accessibility/availability, empathy, and care/safety (see table 1).

**Table 1:** Types of complaints made by service users in health facilities: findings from the questionnaire survey

Categories	Example
Environment/equipment	• No water supply, poor hygiene and no toilets in health facilities. • No waiting room, no furniture and poor infrastructure.
Accessibility/ availability	• Health facility opens late and closes early, health facilities closes at 2 pm. • Medicine not available, medicine not given, all medicine should be free, need to pay for medicine. • Service providers are not regular, service providers do not come to office on time, no doctor. • There should be a 24-hour service, service should be available during the weekend. • No timely service, delay in providing services. • Need of diagnostic services such as laboratory and X-ray. • Immunization service and PHC out-reach clinic not managed well or not near to their residence. • No ambulance, no delivery service. • Maternity incentive not given, or delayed, to mother.
Empathy (respect/dignity/staff attitudes)	• Service providers behave rudely. • Treatment information, not adequately provided. • Service providers not responsive. • No confidentiality.
Care/treatment /safety	• Medicine did not work, with poor quality. • Medicine near to expiry or expired. • Misuse of medicine by service providers.

Although the above findings indicates that many individual-level complaints are related to broader system level issues, some believed that complaints made by service users were primarily intended to address their individual needs and problems, and were not related to the broader health service and issues related to PHC system management overall. Furthermore, complaints made were sometimes seen as irrelevant:

‘Well, complaints made by individuals are related to their personal urgent problems and are not directly program related. For example, if an individual comes to have his tooth extracted but does not get that service, then he complains about it, which is related to his individual problem’ (Qualitative interview, clinic manager, health post1).

An interesting finding, while interviewing the district-level participants, was that there were complaints coming from the community directly to the district level. This made the District Public Health Office chief very busy, and many of these complaints could have been addressed locally. There was no apparent difference in the types of complaints appearing at both levels.

There was also no proper system for recording complaints and analyzing them at either the district or health facility levels. Qualitative interviews revealed that the District Public Health Office chief had a practice of maintaining a personal diary to record the complaints and there was no official recording system in place. In the case of the health facilities, the clinic manager generally managed complaints but, again, there was no system for recording these. Similarly, when complaints were taken to village HFMC meetings, there was a practice of discussing these and making decisions, but there was no documentation in meeting minutes.

### The channels of complaints

Qualitative interviews found that the service users favored informal approaches over formal channels. Informal approaches included putting complaints verbally in person or by phone, whereas formal approaches were in writing such as via the government mandated suggestion boxes.

‘Complaints are mainly made verbally. If not satisfied, they come to us [HFMC] […] and share their grievances, but there is no practice of putting complaints in written form (Qualitative interview, HFMC member, health post1).

It was found that service users placed complaints in the suggestion boxes very rarely, because they did not know about their existence, know how to make complaints, or thought it would take a long time to get a response. In many clinics, there was no suggestion box, and at other times the suggestions were perceived as irrelevant.

‘… not only at the health facility level but even at the district level, the situation is that suggestion boxes are filled up with ‘spider webs’. As far as I know the suggestion box is not in use. No one puts their complaints or suggestions [into the suggestion box] by writing onto a piece of paper. Many do not know about its existence. So I do not see any importance of it. (Qualitative interview, clinic manager, PHC centre2).

The findings of the qualitative interviews were corroborated by the quantitative survey results. Among the small proportion of service users who ever made complaints (*n* = 20), nineteen reported complaining directly to service providers. HFMC members (*n* = 89) also reported that complaints were put directly to: HFMCs (75%), FCHVs (63%) and service providers (42%), with only rarely being lodged via the suggestion box (2%). Similarly, service providers (*n* = 67) opined that complaints were put directly to service providers (84%), and formal channels such as the suggestion box were rarely used.

Different factors emerged as reasons for choosing a particular route for complaining. The main reason for using informal approaches is the lack of an established culture of using formal written channels and the suggestion boxes.

‘In current practice, if they [public] have anything inside, they put it directly [to the service provider], but there is no practice of putting the suggestions in the suggestion box by writing. In fact the public have no habit of making complaints using such a channel’ (Qualitative interview, auxiliary nurse midwife, health post2).

Another reason for choosing one approach for complaining over others was approachability or accessibility. Those who could speak to service providers made complaints directly either in-person or by phone. Others who felt they could not speak directly to service providers, made complaints indirectly via HFMCs, FCHVs, or by sharing with their community leaders.

Similarly, FCHVs in the focus group also mentioned that many ordinary people in villages, especially women, made complaints through them because FCHVs are accessible and approachable to them.

‘If the health facility is not opened on time or if the staff are not available, then the public who know us come to us and complain about it. Then we raise this issue in our monthly meeting with staff, asking staff why such a thing happened. Each FCHV puts the grievances coming from village members to the staff. The public tell us about their complaints when they meet us in the village, in the mother’s group meetings or somewhere else in informal settings. For them we are easy to reach and approachable’ (Focus group, FCHV group).

Another factor was the need for a prompt response:

‘The public often have an urgent need to talk so they do not wait, but make complaints directly by meeting us or by phone. If they use the suggestion box, it takes a long time [to get a response]’ (Qualitative interview, HFMC chair, PHC centre1).

Many respondents also felt that service users preferred to use direct ways of making complaints due to their perceived effectiveness in making the service provider accountable. However, such direct approaches were sometimes reported as confrontational.

The choice of channel also depended on the types of complaints made. If the problems were not solved by the clinic manager, or the complaint was related to management and the clinic manager, respondents tended to make complaints to the HFMC.

### Reasons for not complaining

Six key themes arose as possible explanations for not making complaints.

1. Lack of channels for making complaints.
  The lack of channels for making complaints, and lack of awareness by service users about the existing complaint mechanisms or how to use them, were a hindrance. The questionnaire survey results showed that only 18% (*n* = 40) of the service users reported ever having heard of the suggestion boxes. Among the 22 health facilities observed, only five were found to have suggestion boxes in place, suggesting that this avenue for complaining was unavailable to service users.
  ‘No, we have not used it so far. I am in confusion as to when and how to use it. It was labelled as a complaint box so I thought that it is used for putting complaints in, but what to write and how to use it is confusing to me. Also, I am afraid that if I put complaints there, someone may notice it and catch me. I am confused whether the procedure is like dropping letter in the mail box or not’ (Focus group, community people-female group).
2. Knowledge of service entitlements
  Lack of information about health facilities and health services was found to be one of the key reasons why service users, especially Dalit, uneducated, and poor, did not make complaints.
  ‘No, we have not voiced our grievances because we do not have general information about what services are available from the hospital [health post]. If there is no available [medicine], they [service providers] advise us to buy it from elsewhere. Then we return right away. If we knew more information about the health post, we might ask questions ‘why we do not get it [medicine or services]?’ We are unaware of all these. Then how can we raise any complaints?’ (Focus group, community people-female group).
  Some interviewees, particularly service providers, also indicated that some of the complaints of service users were irrelevant, arising due to unrealistic expectations of the health facility.
3. Power differential between service providers and service users
  Power differentials was identified as a common cause for not complaining. The general public looked up to service providers and perceived them as high salary, respected people in society. For the general public, all the health workers are considered to be doctors (in fact, whereas, few PHC service providers are doctors), who hold positions with high social status in the Nepalese community. Apart from this, they were afraid that making complaints may cause service providers to get angry or to deny them good quality health services the next time they visited.
4. Lack of possibility to choose alternative providers
  Closely linked with the above context was a lack of ‘exit’ options (e.g. to alternative providers), which compelled service users to stay silent, even when they were not happy with the local PHC services. Many services in the public health facilities were free, and these were also the most geographically accessible clinics, being located in the villages. Apart from this, some of the public health programs such as immunization, and Vitamin A distribution, were only available from the public health facilities and not from alternative providers. Service users did not want to make health service providers unhappy by making complaints. One NGO staff member who was working with the district health system and local community to strengthen the community’s voice commented:
  ‘How can Dalit, women, and the marginalized speak their minds with service providers? They think what the government does is all right. Health is the matter related to life and death. If you or your family member becomes ill, you have to go to the same place. Then, how could you take issue with the service providers? In villages, there is no option’ (Qualitative interview, staff, NGO).
5. Lack of established culture of questioning providers
  Many respondents reported that there was no culture developed among service users to ask ‘why’ when things were perceived as inadequate. There was an attitude of accepting ‘business as usual as being okay’ among service users (Qualitative interview, Dalit HFMC member 1, sub-health post1).
  A service provider commented,
  ‘If service providers say that this service is not available today, they [service users] just return, but there is no culture of questioning the service providers as to “why”’ (Qualitative interview, auxiliary health worker, health post1).
6. Perceived lack of responsiveness
  Interviewees suggested that service users also did not complain because they had a feeling that there would be no response from service providers to the complaints made.
  ‘Well, I think people do not make complaints because they might think it is useless to talk about such grievances [with service providers] because there would be no solution’ (Qualitative interview, office assistant, sub-health post1).

## Discussion

The main aim of this study was to understand the complaint handing process in a rural and remote PHC setting of the Dang District of Nepal. To the best of our knowledge, this is the first comprehensive study internationally which has studied various aspects of complaint procedures in a primary health care context in a low income setting. Strengths of the study include data being collected from respondents with different backgrounds, varied geographies, different levels of health facilities, and multiple roles (e.g. service users, HFMC members, frontline service providers, district level managers and local service providers, and NGO members). The study did have some limitations. As there was no proper system of recording how many complaints were lodged, it was not possible to collect detailed information about the types or frequency of complaints. However, the use of different data sources did help to address this limitation. All the survey and many qualitative participants were interviewed in the health facility environment which could have led to a sense of restraint. However, every effort was made during the interviews to facilitate a rapport with respondents, and to explain the purpose of the research and ensure the de-identified nature of participants’ responses. Out of the 39 individual qualitative interviews, only five were women. Although there was a good proportion of women service providers and HFMC members, key positions within the health facilities and HFMCs were mostly occupied by men. As our criteria for selecting qualitative interview participants were guided by the principle of selecting “information-rich cases” [43] to yield in-depth understanding on the issues under study, they were our key informants resulting in the participants’ gender imbalance. However, when possible we tried to include female members from HFMCs and service providers to ensure “heterogeneity” [43] and include diverge perspectives. Furthermore, in the case of focus groups with service users, we considered more female groups than male.

There were few complaints coming to the PHC system of the Dang District. Service users tended to make complaints only when they considered a problem to be very severe. Generally, those who were educated and financially secure lodged complaints, much less so the poor and Dalit. Ironically, the latter groups were the ones who use PHC services the most. Service users favored informal approaches, such as making complaints verbally in person or by phone, over formal approaches such as writing. Many studies in health and non-health contexts have shown that only a portion of those with grievances express them and most of, complainants adopt an informal approach [44–47]. A national survey conducted in Israel showed that, out of 1500 participants, 382 (25.5%) mentioned that they had a grievance but did not complain, and only 143 (9.5%) had actually complained to their service providers. There, three quarters of the complainants submitted their complaints verbally [46].

The main types of complaints found in our study were related to environment/equipment, accessibility/availability, empathy and care/safety. When compared with Donabedian’s quality of care model of structure, process and outcome, most of the complaints fell into the structure and process domains [48].

Many factors emerged as reasons for not complaining. The service users were unaware of the service entitlements. Without adequate information about the health facilities and services, it is very unlikely service users will be able to voice their suggestions for improving the health system. Lack of exit options appeared to have compelled service users to stay silent and loyal even when they were not happy with the PHC services. This finding is contrary to Hirschman’s original conceptualization which was that, when people do not have exit options, they are likely to voice their concern about the system [47, 49].

Perhaps the reason for this apparent difference is that there is lack of an established culture for service users to raise questions when something goes wrong in the system. This can be attributed to the low level of empowerment. It may also result from the PHC system in Nepal providing basic PHC services. Hence, the issues or problems in this PHC system may not be perceived as serious compared to the problems people may encounter in larger hospitals.

A number of policy and practical implications arise from the findings of the study. These include the following.

1. Improved knowledge of service entitlements
  Information is a precondition to voice [50, 51]. Information empowers service users to put complaints strongly and appropriately. Hence, it is necessary to put an effort into raising the awareness of service users about health facilities, health services and peoples’ entitlements. There is also a need to raised awareness of the purpose and use of complaints channels, so that services users are empowered to make complaints.
2. Orientation of health workers
  Due to the ubiquity of informal complaints, and many of these coming to service providers in person, most of the complaints occurred outside the purview of formal recording and reporting procedures. Hence, there is a need for proper dialogue and trust between service uses and service providers to address the complaints of service users. For this, front line service providers need proper orientation into complaints handling and about the importance of community voice to strengthen the health system.
3. Establish proper complaints systems
  The Dang PHC system was missing important information and feedback from its users which could be useful to improve the quality of health services. First, there should be proper channels for complaints to be made [24, 52] along with a system for registering complaints. Many participants in this study shared negative ‘word of mouth’ complaints with friends and other people in the community. Hence, it would be useful to channel their grievances (hidden complaints) to the health system appropriately. Others appeared to voice complaints informally through channels such as HFMC members, FCHVs and directly to health workers. Use of formal channels such as suggestion boxes or written complaints was almost non-existent. Taking into consideration the socio-cultural context, this suggests it is necessary to promote such informal channels. Promotion of channels such as HFMC members and FCHVs for receiving community concerns is likely to be crucial for those who are not able to speak directly to service providers or who have problems with literacy. As mentioned elsewhere in this paper, HFMCs have representation from different village community groups and similarly FCHVs are health volunteers from local villages and are selected from mothers’ groups [28]. FCHVs are supposed to meet with service providers at monthly meetings at health facilities. As FCHVs are proven for delivering community based health services in the Nepalese context [53], this study also identifies their potential for channelling complaints of the local villagers to the health system. Hence, strengthening of this community health system (FCHVs, with their mothers’ groups networks and HFMCs) for strengthening the complaint system seems worthwhile. However, concurrently strengthening formal channels such as written complaints and the suggestion boxes is also necessary.
  Second, there is need for a complaint management system. For this, proper recording of the complaints made at or about the health facilities is necessary to ensure that understanding about the frequency and nature of complaints is both improved and addressed [7, 46]. Such a record (e.g. a register) would also give an idea of which mechanisms are more appropriate and effective. There should be clear responsibility conferred to a person responsible for hearing complaints in health facilities. Service providers and HFMCs at health facilities need to work together for appropriate complaint management.
  Finally, complaints were also coming to the District Public Health Office authority so it is necessary to give more authority to local levels, especially HFMCs at health facilities. Service users should be able to have trust in the capacity of such local authorities to address their complaints. The complaints coming to the local health facilities should be communicated to the district level so that necessary support from the district level can be sought [46]. Furthermore, when service users are not satisfied with the response by complaining to frontline service providers, there is a need of provision for service users to directly report complaints to district health managers [24, 54].
  There are a number of areas for future research. As this study found community members preferred informal over formal complaints channels, a future study could shed light on the relative effectiveness of these channels to address the community’s concerns. The study found that citizens preferred to stay silent, instead of challenging health system problems by voicing concerns when they were not happy with the services due to absence of exit options. More in-depth study would be useful to understand the dynamics of exit, voice, and loyalty [55] in the health care context of Nepal and similar settings. Future research also needs to consider the service providers’ responsiveness to the complaints lodged in the PHC system. A quality of care model could be useful to help categorize complaints. One such is Donabedian’s structure, process and outcome model [48]. Although it was beyond the scope of the present study, such a quality of care model could be used as a guiding framework for complaints in future studies.

## Conclusion

Very few service users made complaints to PHC services in Nepal. The low level of complaints in Nepal’s PHC system does not, however, imply that service users did not have grievances with the system. In fact, service users did have unexpressed grievances. As such, this study indicates that the Nepalese health system is losing valuable information from its communities, leaving the system with a weaker basis from which to improve health service quality [46]. Several contextual factors related to community and the health system were identified as reasons for not complaining. There is a need to increase awareness among service users about service entitlements and complaint mechanisms, and to establish proper complaints registers and feedback mechanisms. An intended outcome of this study is that it will help further development of mechanisms for an effective complaint handling system and, in turn, improve the quality of care of PHC services in Nepal and other LMICs with similar contexts.

## Abbreviations

FCHV: Female community health volunteer
HFMC: Health facility operation and management committee
NGO: Non-government organization
PHC: Primary health care
